# Citrulline Supplementation Improves Organ Perfusion and Arginine Availability under Conditions with Enhanced Arginase Activity

**DOI:** 10.3390/nu7075217

**Published:** 2015-06-29

**Authors:** Karolina A.P. Wijnands, Dennis M. Meesters, Kevin W.Y. van Barneveld, Ruben G.J. Visschers, Jacob J. Briedé, Benjamin Vandendriessche, Hans M.H. van Eijk, Babs A.F.M. Bessems, Nadine van den Hoven, Christian J.H. von Wintersdorff, Peter Brouckaert, Nicole D. Bouvy, Wouter H. Lamers, Anje Cauwels, Martijn Poeze

**Affiliations:** 1 Department of Surgery, NUTRIM School for Nutrition, Toxicology and Metabolism, Maastricht University Medical Center, Maastricht 6200 MD, The Netherlands; E-Mails: d.meesters@maastrichtuniversity.nl (D.M.M.); k.vanbarneveld@maastrichtuniversity.nl (K.W.Y.B.); r.visschers@maastrichtuniversity.nl (R.G.J.V.); hmh.vaneijk@maastrichtuniversity.nl (H.M.H.E.); imnoteviliam@hotmail.com (B.A.F.M.B.); n.vandenhoven@student.maastrichtuniversity.nl (N.H.); c.von.wintersdorff@mumc.nl (C.J.H.W.); n.bouvy@mumc.nl (N.D.B.); m.poeze@maastrichtuniversity.nl (M.P.); 2 Department of Toxicogenomics, GROW School for Oncology and Developmental Biology, Maastricht University Medical Center, Maastricht 6200, The Netherlands; E-Mail: j.briede@maastrichtuniversity.nl; 3 Department of Molecular Biomedical Research, VIB, Ghent B-9000, Belgium; E-Mails: benjamin.vandendriessche@dmbr.ugent.be (B.V.); Peter.Brouckaert@dmbr.vib-ugent.be (P.B.); anje.cauwels@vib-ugent.be (A.C.); 4 Department of Biomedical Molecular Biology, Ghent University, Ghent B-9000, Belgium; 5 Department of Anatomy & Embryology, Maastricht University Medical Center, Maastricht 6200, The Netherlands; E-Mail: wh.lamers@maastrichtuniversity.nl

**Keywords:** arginase, arginine, citrulline, microcirculation, nitric oxide

## Abstract

Enhanced arginase-induced arginine consumption is believed to play a key role in the pathogenesis of sickle cell disease-induced end organ failure. Enhancement of arginine availability with l-arginine supplementation exhibited less consistent results; however, l-citrulline, the precursor of l-arginine, may be a promising alternative. In this study, we determined the effects of l-citrulline compared to l-arginine supplementation on arginine-nitric oxide (NO) metabolism, arginine availability and microcirculation in a murine model with acutely-enhanced arginase activity. The effects were measured in six groups of mice (*n* = 8 each) injected intraperitoneally with sterile saline or arginase (1000 IE/mouse) with or without being separately injected with l-citrulline or l-arginine 1 h prior to assessment of the microcirculation with side stream dark-field (SDF)-imaging or *in vivo* NO-production with electron spin resonance (ESR) spectroscopy. Arginase injection caused a decrease in plasma and tissue arginine concentrations. l-arginine and l-citrulline supplementation both enhanced plasma and tissue arginine concentrations in arginase-injected mice. However, only the citrulline supplementation increased NO production and improved microcirculatory flow in arginase-injected mice. In conclusion, the present study provides for the first time *in vivo* experimental evidence that l-citrulline, and not l-arginine supplementation, improves the end organ microcirculation during conditions with acute arginase-induced arginine deficiency by increasing the NO concentration in tissues.

## 1. Introduction

Sickle cell disease can be affected by acute life-threatening complications, such as recurrent vaso-occlusive events, pulmonary hypertension and severe hemolysis [1,2]. In an acute crisis with intravascular hemolysis [3,4], arginase is released in large quantities by damaged red blood cells [1,4,5]. This arginase rapidly consumes the locally-available arginine, leading to a diminished production of the main vasodilator of the microcirculation, nitric oxide (NO) production [6], which contributes to an impaired microvascular flow, resulting in end-organ damage [1,2]. Therefore, arginase-induced arginine deficiency is believed to play a key role in the pathogenesis of sickle cell disease-induced end-organ failure [2,7,8]. Other mechanisms possibly contributing to the decreased microvascular flow are increased oxidative stress and the release of hemoglobin from the damaged red blood cells, which consumes NO [3,4]. Therefore, supplementing arginine to restore the depleted arginine pools was suggested to be a good therapeutic approach to treat end-organ damage in sickle cell disease [9].

Previous studies with l-arginine supplementation in transgenic sickle cell mice showed a reduction in oxidative stress and hemolysis and increased NOx concentrations [10,11]. However, clinical results using l-arginine were less consistent. In a non-controlled patient study, l-arginine supplementation reduced pulmonary pressure, but also tended to further increase arginase activity [5]. Another study similarly found that l-arginine supplementation increased arginase activity, but found no increase in NOx concentrations [7]. A third study showed highly variable changes in exhaled NO concentrations during l-arginine supplementation, while no effects on clinical parameters were found [12]. Finally, in a study comparing l-arginine with a phosphodiesterase inhibitor, no clinical benefits were found during l-arginine supplementation [13], suggesting no extra NO production. In these patients, ornithine concentrations increased significantly, suggesting an enhanced arginase activity, while citrulline concentrations were unchanged.

Instead of l-arginine, its precursor, l-citrulline, which is endogenously produced in the gut [14,15], may be the preferred substrate to enhance intracellular arginine availability by enhancing arginine *de novo* synthesis and NO production in pathophysiological conditions with increased arginase activity, resulting in enhanced arginine consumption, such as sickle cell disease [16] or endotoxemia [17]. As for endotoxemia, another condition with high arginase activity, l-citrulline supplementation resulted in increased NO production and better microvascular flow than l-arginine supplementation [17]. In sickle cell disease, only one non-controlled pilot study showed that citrulline supplementation enhanced the arginine availability and relieved fatigue and dyspnea in these patients [16]. However, the effects of l-citrulline supplementation on the microcirculatory flow and tissue NO production have not been determined or compared to that of l-arginine supplementation in a preclinical setting in experimental conditions with an acute increase of arginase [16,18]. We, therefore, prior to a clinical study, determined the effect of l-citrulline compared to l-arginine supplementation on arginine-NO metabolism, arginine availability in blood, kidney, liver and jejunal tissue and the microcirculation in the jejunum of mice with an acutely-enhanced circulating concentration of arginase.

## 2. Material and Methods

### 2.1. Animals

Forty-eight male C57BL/6J mice (25–30 grams) were bred at the Department for Molecular Biomedical Research of Ghent University. Mice were individually housed and subjected to standard 12-h light-dark cycle periods. Mice were fed standard lab chow and water *ad libitum*. Mice adapted to the laboratory environment for 7 days prior to the start of the experiments. The study protocol was approved by the Committee on the Ethics of Animal Experiments of Ghent University (approval number EC2012-028).

### 2.2. Experimental Protocol

This experimental model, with an acute arginine-deficient state induced by intraperitoneal (i.p.) arginase injection, was developed to determine the preferential substrate, l-citrulline or l-arginine supplementation, on arginine availability, tissue NO production and microcirculation. The arginase concentration used in this study, 1000 IE/25 g, was based on previous experience in our group, resulting in a >60% reduction in plasma arginine concentrations [19], and on clinical studies in which plasma arginase activity during the steady-state phase of sickle cell disease caused a ~1.5-fold decrease in plasma arginine concentrations [4,20].

**Figure 1:**
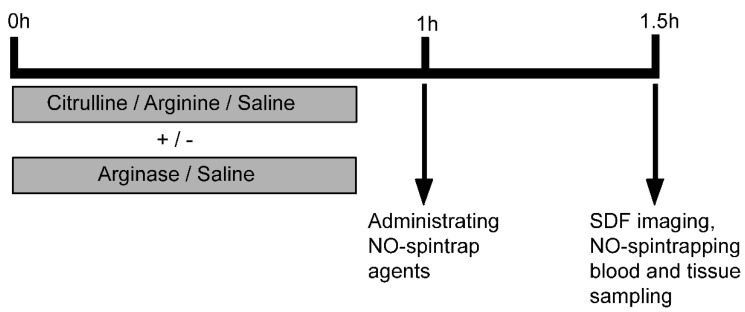
Experimental setup of the acute arginase model. Mice received an intraperitoneal injection with sterile saline or arginase combined with l-citrulline or l-arginine at time zero (*t* = 0 h). After 1 h (*t* = 1 h), mice received either spin-trap agents to measure the nitric oxide (NO) production *in vivo* or a sterile saline injection as placebo treatment. After 1.5 h (*t* = 1.5 h), side stream dark-field (SDF) imaging was used to quantify the microcirculation in the jejunal villi or organs were harvested to determine the formed iron-diethyldithiocarbamate (DETC) complexes as a parameter for the NO production *in vivo*. At the end of the experiment, blood and tissue samples were harvested for amino acid determination.

At 0 h, mice were injected intraperitoneally with sterile saline (NaCl 0.9%; 0.5 mL), l-citrulline (Cit; 37.5 mg; 0.2 mmol) or l-arginine (Arg; 37.5 mg; 0.2 mmol), with or without arginase in a separate syringe and on the contralateral site injected (Sigma, St. Louis, MO, USA, 40 IE/g protein, 0.1 mL). Five groups were studied: control (*n* = 8), arginase injection (*n* = 8), citrulline supplementation (*n* = 8), arginine supplementation (*n* = 8), citrulline + arginase (*n* = 8) and arginine + arginase (*n* = 8) (See Figure 1 for the experimental setup). After the injection, food was withheld, but water was provided throughout the experimental phase. One hour after the i.p. injections, spin trap agents were administered i.p. and subcutaneously (s.c.) for tissue NO production measurements (*n* = 3 per group), as described below. Mice used for microcirculatory measurements received an equal amount of sterile saline. Simultaneously, mice were premedicated with 0.01 mg/kg Temgesic^®^ (Reckitt & Colman Products LTD., Kingston-Upon Hill, U.K.) s.c.

One and a half hours after the initial injection, anesthesia was induced with 4% isoflurane (Abbott Laboratories LTD, Maidenhead, U.K.). During the measurements of the tissue NO production and microcirculatory analysis in the jejunal mucosa with a side stream dark-field (SDF) imager, anesthesia was maintained with 2% isoflurane. All imaging experiments were done by an experienced investigator, who was blinded to the treatment allocation. Images were analyzed by 2 independent, experienced researchers, both blinded to the treatment.

Throughout the experiment, body temperature was maintained at 37 °C, using an infrared heating lamp with a temperature controller connected to a rectal probe. At the end of the experiment, blood was sampled via cardiac puncture, after which the animals were euthanized by cervical dislocation.

### 2.3. Amino Acid Analysis

**Figure 2:**
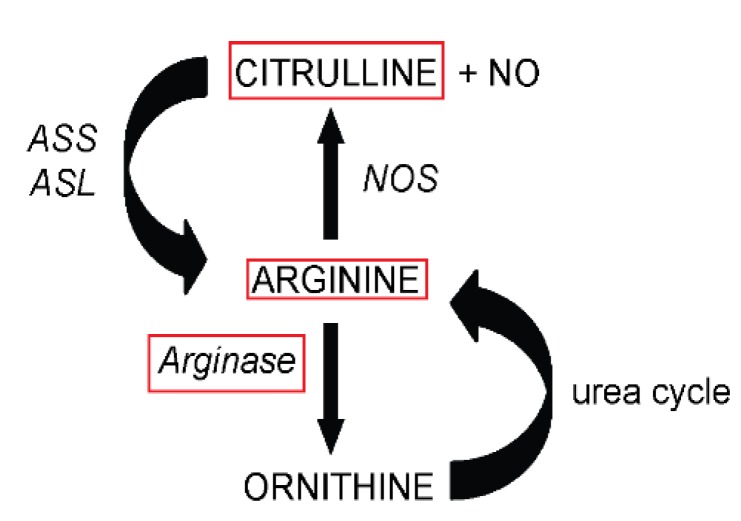
Schematic overview of the relevant metabolic pathways of arginine. Abbreviations: NO, nitric oxide; NOS, nitric oxide synthase; ASS, argininosuccinate synthase; ASL, argininosuccinate lyase.

After deproteinization, plasma and tissue (jejunum, liver and kidney) amino acid concentrations were determined with a fully-automated liquid chromatography-mass spectrometry system (LC-MS, Thermoquest LTQ, Veenendaal, The Netherlands) as described before [17,21]. Jejunal, liver and kidney tissue were used to determine the role of the gut-liver-renal axis in an acute arginine-deficient state. To determine the arginine availability in plasma and tissue, the arginine availability index (AAI) ((arginine)/((ornithine) + (lysine))) was calculated. This index is based on the uptake in cells of arginine, ornithine and lysine by the y^+^ transporter system and provides an indication of the relative available arginine for metabolic pathways [22]. The arginine transporter y^+^ consists of four different types of cationic amino acid transporters (CATs); CAT-1, CAT-2a and CAT-2b, CAT-3 and CAT-4 [23,24]. Only CAT-1 and CAT-2b have a high affinity for cationic amino acids, such as arginine and citrulline; therefore, the influence of CAT-2a, CAT-3 and CAT-4 is negligible in this study. CAT-1 is expressed in almost all adult cells during physiological conditions [25], whereas CAT-2b is only expressed after induction with cytokines or lipopolysaccharide (LPS) treatment in inflammatory cells [23,26], which was not present in this study. Figure 2 provides a schematic overview of the relevant metabolic pathways of arginine.

### 2.4. In Vivo Tissue NO Measurements

The *in vivo* NO production in jejunum, liver and kidney was determined in tissue samples of mice (*n* = 18) injected with spin-trap agents, as described previously [17]. Briefly, mice were injected s.c. in the scruff of the neck with a mixture of FeSO_4_·7H_2_O (37.5 mg/kg), sodium citrate (190 mg/kg) and i.p. with diethyldithiocarbamate (DETC, 500 mg/kg) 30 min prior to sacrifice [27]. NO is trapped with Fe^2+^-dithiocarbamate, an mono-nitrosyl iron complex (MNIC), and measured with electron spin resonance (ESR) spectroscopy, as described [17,27]. NO concentrations were calculated from the height of the three-line NO amplitude using Bruker WINEPR software [17,27].

### 2.5. Jejunal Microcirculation Measurements with SDF Imaging

Microscopic visualization of the intestinal mucosal microcirculation in the jejunal villi with the SDF-imager (Microscan, Amsterdam, The Netherlands) [28,29] was described in detail [17] and chosen because the gut is easily accessible and a frequently affected end organ [30]. In brief, a small incision was made in the jejunum to visualize the jejunal villi. A specially-designed stand was used to stabilize the SDF-imager and to avoid pressure on the jejunal villi during the measurements. The microcirculation, determined as the total number of perfused vessels per villus, was analyzed using Automated Vascular Analysis software 3.0 (Microscan, Amsterdam, The Netherlands), adjusted according to De Backer *et al.* [31,32,33]. Furthermore, the average microvascular flow index (MFI), a semiquantitative assessment of the predominant type of flow in the villi, was determined in the four quadrants of the image (0 = absent, 1 = intermittent, with at least 50% of the time having no flow, 2 = sludging, 3 = normal or 4 = hyperdynamic flow) [32]. All imaging experiments were performed by an experienced investigator, and images were analyzed by two independent, blinded, experienced researchers.

### 2.6. Statistical Analysis

Statistical analysis of the data was performed using SPSS 19.0 (SPSS, Chicago, IL, USA). In the experiment, comparisons were made between control and arginase groups to test the effect of arginase treatment. The control group was also compared with the citrulline- and arginine-supplemented groups to determine the effect of l-citrulline or l-arginine supplementation during the control condition. Finally, the citrulline + arginase group and the arginine + arginase group were compared with the arginase group to test the effect of l-citrulline or l-arginine supplementation during an acute arginine-deficient state. In case of a Gaussian distribution, one-way ANOVA with *post hoc* Bonferroni correction between groups was used. A two-sided *p* < 0.05 was considered as statistically significant. Data are represented as the mean and standard error of the mean (SEM).

## 3. Results

### 3.1. Improved Plasma Amino Acid Concentrations after Citrulline Supplementation in Arginase-Treated Animals

**Figure 3:**
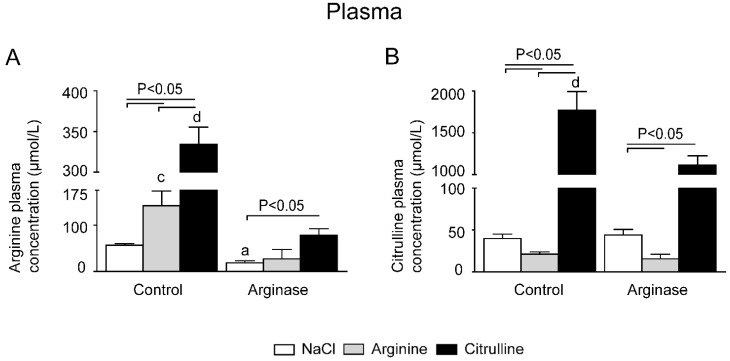

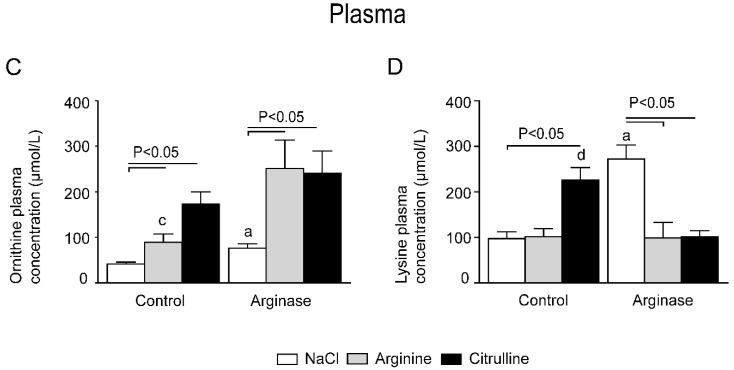
Effect of l-arginine or l-citrulline supplementation on plasma amino acid concentrations in control and arginase treated mice. (**A**) Plasma arginine concentrations during basal and arginase-treated conditions with or without citrulline or arginine; (**B**) plasma citrulline concentration in all treated groups; (**C**) ornithine concentrations during basal and arginase-treated conditions; (**D**) lysine concentrations in plasma were measured with HPLC. Plasma concentrations are displayed as μmol/L. Significance: ^a^ *p* < 0.05 *vs.* control; ^b^ *p* < 0.05 *vs.* arginine; ^c^ *p* < 0.05 *vs.* arginine + arginase; ^d^ *p* < 0.05 *vs.* citrulline + arginase.

Arginase injection resulted in a <35% reduction of plasma arginine concentrations compared to control animals (*p* < 0.0001; Figure 3A; see Supplementary Table S1 for normalized values of all amino acids). As expected, both l-arginine and l-citrulline supplementation resulted in higher plasma arginine and citrulline concentrations during control conditions (Figure 3A,B). l-citrulline supplementation mediated a >4-fold significant increase in plasma arginine concentration in arginase-treated animals, whereas arginine supplementation was unable to mediate a significant increase in plasma arginine concentration in arginase-injected animals (Figure 3A).

In parallel to the decreased plasma arginine concentrations in arginase-treated animals, ornithine concentrations were significantly higher (Figure 3C), indicating the conversion of arginine to ornithine by the injected arginase. Both l-citrulline and l-arginine significantly increased ornithine concentrations in arginase-treated animals. Interestingly, citrulline concentrations were significantly lower in arginase-treated animals supplemented with l-arginine than in animals treated with arginase alone (Figure 3B).

### 3.2. Depleted Tissue Amino Acid Concentrations Were Restored by Citrulline and Arginine Supplementation

**Figure 4:**
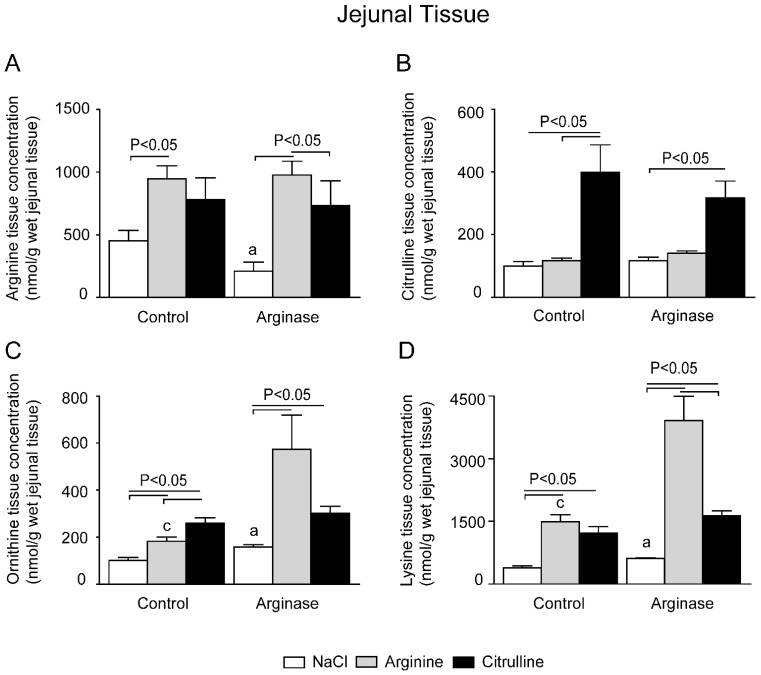
Effect of l-arginine or l-citrulline supplementation on jejunal tissue amino acid concentrations in control and arginase-treated mice. Jejunal tissue concentrations during basal and arginase-treated conditions of (**A**) arginine, (**B**) citrulline, (**C**) ornithine and (**D**) lysine were measured with High Performance Liquid Chromatography (HPLC). Tissue concentrations are displayed as nmol/g wet tissue). Significance: ^a^ *p* < 0.05 *vs.* control; ^b^ *p* < 0.05 *vs.* arginine; ^c^ *p* < 0.05 *vs.* arginine + arginase; ^d^ *p* < 0.05 *vs.* citrulline + arginase.

**Figure 5:**
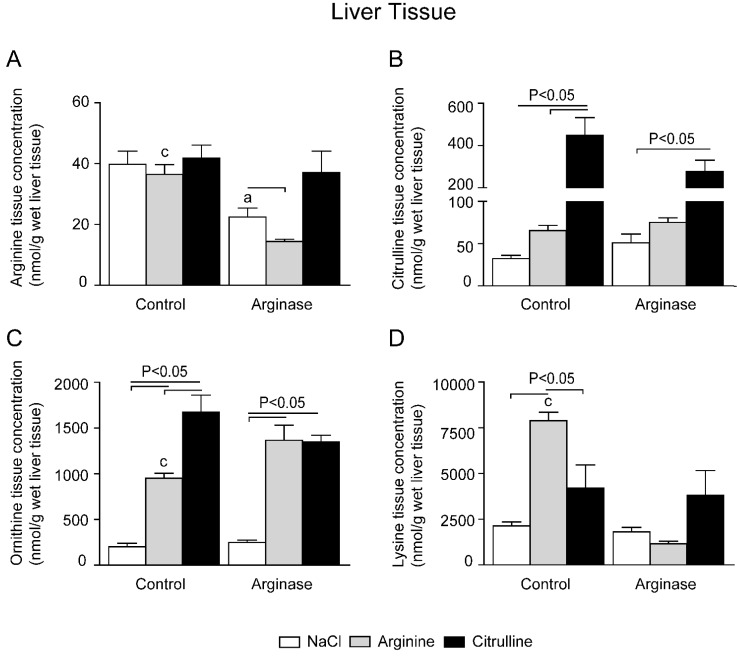
Effect of l-arginine or l-citrulline supplementation on liver tissue amino acid concentrations in control and arginase-treated mice. Liver tissue concentrations during basal and arginase-treated conditions of (**A**) arginine, (**B**) citrulline, (**C**) ornithine and (**D**) lysine were measured with HPLC. Tissue concentrations are displayed as nmol/g wet tissue). Significance: ^a^ *p* < 0.05 *vs.* control; ^b^ *p* < 0.05 *vs.* arginine; ^c^ *p* < 0.05 *vs.* arginine + arginase; ^d^ *p* < 0.05 *vs.* citrulline + arginase.

**Figure 6:**
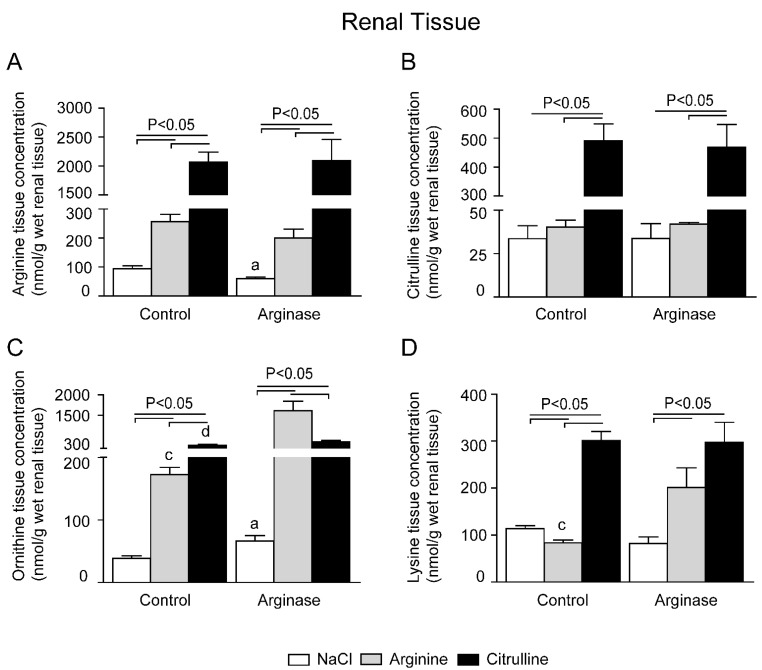
Effect of l-arginine or l-citrulline supplementation on renal tissue amino acid concentrations in control and arginase-treated mice. Renal tissue concentrations during basal and arginase-treated conditions of (**A**) arginine, (**B**) citrulline, (**C**) ornithine and (**D**) lysine were measured with HPLC. Tissue concentrations are displayed as nmol/g wet tissue). Significance: ^a^ *p* < 0.05 *vs.* control; ^b^ *p* < 0.05 *vs.* arginine; ^c^ *p* < 0.05 *vs.* arginine + arginase; ^d^ *p* < 0.05 *vs.* citrulline + arginase.

Next, we investigated the tissue amino acid concentrations in gut, liver and kidney (Figure 4, Figure 5 and Figure 6). These organs play an important role in arginine and citrulline metabolism and are suggested to be involved in sickle cell-induced end organ failure. After arginase infusion, the intestinal arginine concentration was significantly lower (~55%) than in control mice (Figure 4A). l-arginine and l-citrulline supplementation resulted in ~5-fold and ~4-fold higher jejunal arginine concentrations, respectively, compared to mice infused with arginase alone (Figure 4A). Citrulline supplementation resulted in a significantly higher tissue citrulline concentration in the citrulline-treated and citrulline + arginase-treated mice (Figure 4B). Furthermore, the intestinal ornithine concentration was higher in arginase-treated than control mice, but also in l-citrulline- and l-arginine-supplemented mice than in control or arginase-treated mice (Figure 4C)

In liver, arginase treatment resulted in a ~50% lower tissue arginine concentration than measured in control mice (Figure 5A). As was to be expected in view of its very high arginase content, liver arginine content is <10% of that in the gut. l-citrulline supplementation did not result in higher tissue arginine concentrations in the liver of control or arginase-treated mice (Figure 5A), although l-citrulline supplementation increased the liver citrulline concentration (Figure 5B). l-arginine supplementation resulted in comparable arginine concentrations in control animals, but in arginase-treated and arginine-supplemented mice, liver arginine concentrations were significantly lower than in mice that were treated with arginase only (Figure 5A). Interestingly, arginase treatment alone was not associated with increased ornithine concentrations in liver tissue, although l-citrulline- and l-arginine-supplemented mice both had significantly higher ornithine concentrations in liver tissue (Figure 5C).

Comparable to the gut and liver, arginase infusion also significantly lowered renal arginine concentrations (Figure 6A) and increased ornithine levels (Figure 6C). l-arginine supplementation increased renal arginine concentrations, both with and without arginase treatment, compared to their respective control conditions (Figure 6A). l-citrulline treatment resulted in increased citrulline concentrations (Figure 6B) and a ~35-fold increase in arginine concentrations, irrespective of the presence of arginase (Figure 6A). Besides the enhanced arginine concentrations, l-arginine and l-citrulline supplementation in arginase-treated mice resulted in significantly higher renal ornithine concentrations; ~25-fold in the arginine + arginase group and ~8-fold in the citrulline + arginase group (Figure 6C). l-arginine supplementation did not result in higher intracellular citrulline concentrations in the kidney.

Together with arginine and ornithine, lysine is one of the major basic amino acids in plasma. Since it is an essential amino acid, changes in concentration only reflect changes in transport and degradation. The lysine concentration in plasma was significantly higher in arginase-infused mice than in control, arginase + arginine- and arginase + citrulline-supplemented mice (Figure 3D). l-citrulline-supplemented control mice also exhibited a significantly higher plasma lysine concentration than control mice (Figure 2D). The lysine concentrations in jejunal tissue increased ~3-fold in arginine- and citrulline-supplemented mice compared to the control (Figure 4D). Lysine increased ~2-fold in arginase-infused mice compared to the control. l-arginine or l-citrulline supplementation in arginase-infused mice resulted in a ~6.5-fold and ~2.5-fold increase in lysine concentration, respectively. Lysine concentration in liver tissue was only significantly enhanced in the arginine-supplemented group compared to the control (Figure 5D). The renal lysine concentration increased ~3-fold in the l-citrulline supplementation group compared to the control (Figure 6D). In contrast, arginine supplementation during control conditions resulted in a significantly decreased lysine concentration in renal tissue compared to control treated animals. l-arginine or l-citrulline supplementation in arginase-infused mice resulted in a ~2.5-fold and ~3.5-fold increased lysine concentration, respectively, compared to arginase-treated mice alone (Figure 6D).

### 3.3. Arginine Availability in Plasma and Tissues of Arginase-Treated Animals

**Figure 7:**
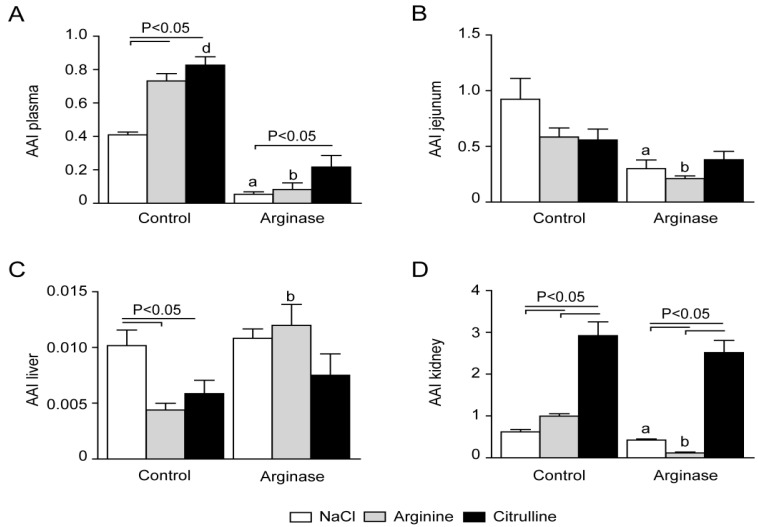
Plasma and tissue arginine availability in control and arginase-treated animals with l-arginine or l-citrulline supplementation. Arginine arginine availability index (AAI; (arginine)/((lysine)+(ornithine))) measured in plasma (**A**), jejunal tissue (**B**), liver (**C**) and renal tissue (**D**) of control and arginase-treated animals with or without supplemented l-arginine or l-citrulline. Significance: ^a^ *p* < 0.05 *vs.* control; ^b^ *p* < 0.05 *vs.* arginine; ^c^ *p* < 0.05 *vs.* arginine + arginase; ^d^ *p* < 0.05 *vs.* citrulline + arginase.

The arginine availability index (AAI) in plasma and tissue ((arginine)/((ornithine) + (lysine))) assumes that the cationic amino acid arginine is transported in and out of cells in exchange for ornithine or lysine [22]. The AAI declined ~8-fold in plasma of arginase-infused and arginase + arginine-supplemented mice and ~4-fold in arginase + citrulline-supplemented mice (Figure 7A). The effects in tissues were less dramatic and had a clear sequence: kidney and jejunum were affected and liver not or even improved. In jejunal tissue, the AAI was significantly reduced in arginase-infused mice compared to the control, which was not enhanced by l-arginine supplementation (Figure 7B). In line with this, l-citrulline supplementation in arginase-infused mice did not result in an enhanced AAI in jejunal tissue of these mice compared to arginase-treated mice alone (Figure 7B). Liver arginine availability was low in all groups, as expected by the high arginase content in liver tissue, and was significantly lowered in the l-arginine and l-citrulline supplementation during control conditions (Figure 7C). l-arginine supplementation resulted in an increased AAI in renal tissue during control conditions (Figure 7D). Arginase infusion resulted in a significant decreased AAI in renal tissue compared to control mice (Figure 7D). This decreased AAI was even significantly more decreased by l-arginine supplementation (Figure 7D). In contrast, l-citrulline supplementation resulted in higher AAI in renal tissue in both control and arginase-treated mice (Figure 7D).

### 3.4. Impaired Tissue NO Production during Acute Arginine Deficiency

**Figure 8:**
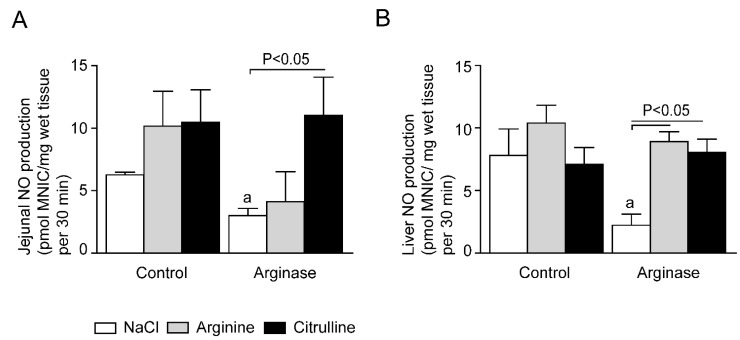

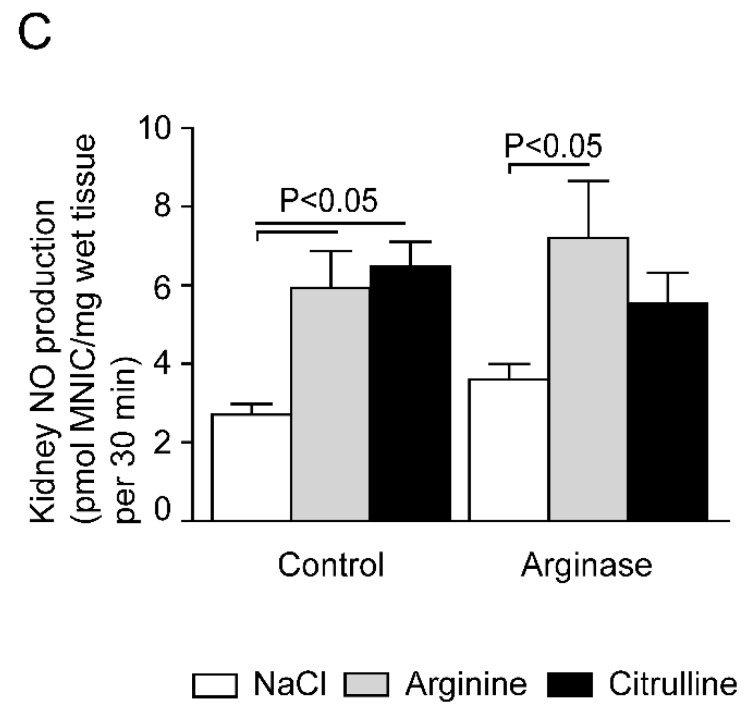
Tissue NO production in control and arginase-treated animals with l-arginine or l-citrulline supplementation. (**A**) The expected arginase-induced decrease in jejunal NO production, determined as pmol mono-nitrosyl iron complex (MNIC)/mg wet tissue, was not present in the arginase + l-citrulline-treated group, whereas in the l-arginine-treated group, the NO production was not enhanced. (**B**) l-citrulline and l-arginine supplementation both resulted in an enhanced NO production in the liver of arginase-treated animals. (**C**) Arginase infusion did not decrease the renal NO production compared to control treated animals. l-arginine supplementation significantly increased renal NO concentration in arginase-treated animals, while l-citrulline only tended to increase the NO levels. Significance: ^a^ *p* < 0.05 *vs.* control; ^b^ *p* < 0.05 *vs.* arginine; ^c^ *p* < 0.05 *vs.* arginine + arginase; ^d^ *p* < 0.05 *vs.* citrulline + arginase.

In line with the decreased arginine availability, the intestinal NO production decreased after arginase infusion (from 6.3 ± 0.2 to 3.0 ± 0.6 pmol MNIC/mg jejunal tissue per 30 min, *p* < 0.001; Figure 8A). l-citrulline supplementation in arginase-treated animals resulted in significantly higher jejunal NO production (*p* < 0.05; Figure 8A). In contrast, arginine supplementation in arginase-treated animals did not result in higher NO production (*p* = 0.2; Figure 8A).

Arginase injection also resulted in a significantly lower NO production in liver (from 7.8 ± 2.1 to 2.2 ± 0.9 pmol MNIC/mg tissue per 30 min, *p* < 0.05, *n* = 3; Figure 8B). l-citrulline and l-arginine supplementation in arginase-treated mice resulted in a significantly higher liver NO production. This NO concentration in the citrulline + arginase group was comparable to citrulline-treated control animals (Figure 8B; not significant, *n* = 3).

Arginase injection did not result in decreased renal NO production (Figure 8C). Interestingly, only l-arginine supplementation could significantly increase renal NO production in arginase-treated animals, while l-citrulline only tended to increase the NO production. In addition, during basal conditions, the NO production in the kidney was significantly higher in both l-citrulline and l-arginine-supplemented mice than control mice (Figure 8C).

### 3.5. Citrulline Supplementation in Arginase-Treated Animals Restored Jejunal Microcirculation

**Figure 9:**
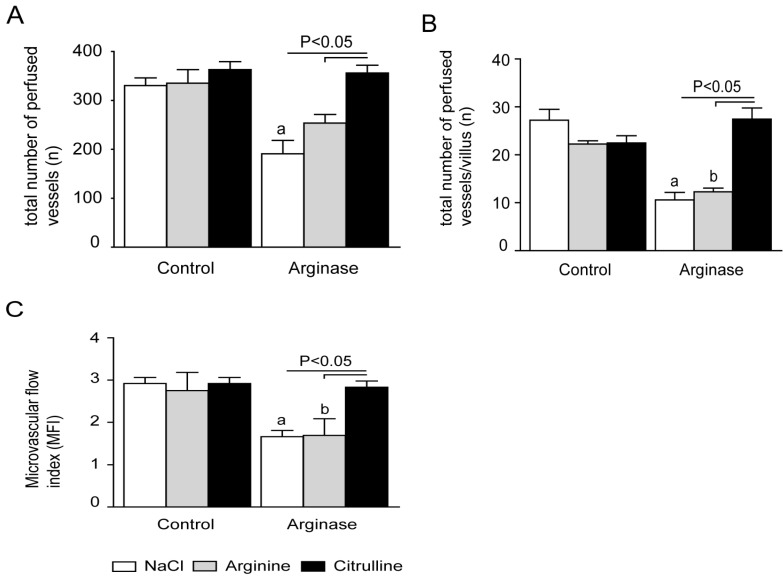
Microcirculatory measurements with side stream dark-field (SDF)-imaging in the jejunal villi. (**A**) The total number of perfused vessels measured with SDF-imaging in the jejunal villi was significantly decreased in arginase-treated mice compared to the control and after l-citrulline supplementation in arginase-treated mice. (**B**) The number of perfused vessels per villus was in line with the total number of perfused vessels, as arginase-treated mice exhibited significantly less perfused vessels per villus compared to control mice. l-citrulline supplementation in arginase-treated mice resulted in an increased number of perfused vessels per villus, whereas l-arginine supplementation did not result in an increased in the number of perfused vessels per villus. (**C**) The microvascular flow index (MFI) was significantly reduced in the arginase- and arginase + arginine-supplemented animals compared to the control and citrulline-treated animals during basal and arginase + citrulline treatment. Significance: ^a^ *p* < 0.05 *vs.* control; ^b^ *p* < 0.05 *vs.* arginine; ^c^ *p* < 0.05 *vs.* arginine + arginase; ^d^ *p* < 0.05 *vs.* citrulline + arginase.

**Figure 10:**
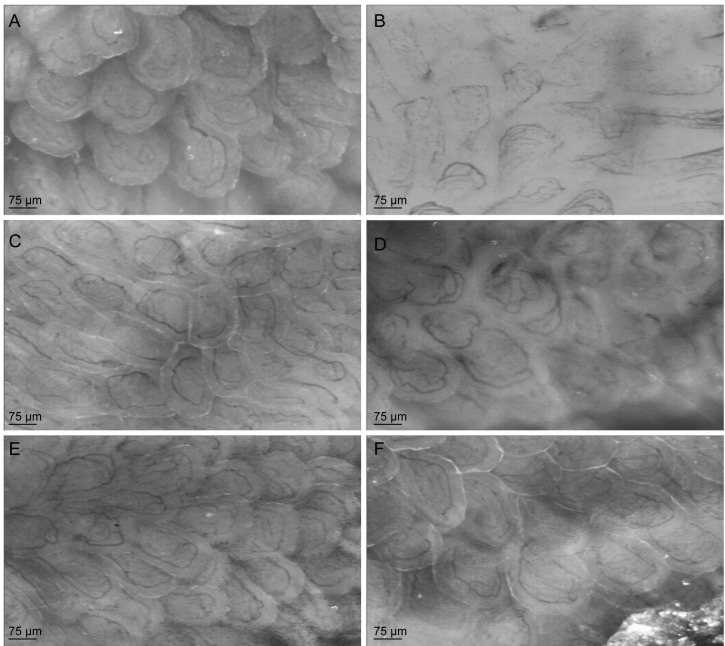
Representative live images of the microcirculatory measurements in jejunal villi with side stream dark-field (SDF)-imaging of control and arginase treated animals with or without l-citrulline or l-arginine supplementation. (**A**) Representative image of the jejunal microcirculation in a control mouse. (**B**) Representative image of an arginase-treated mouse, with a decreased number of perfused vessels per villus. (**C**) Representative image of the jejunal microcirculation in an l-arginine-treated mouse, which shows a comparable perfusion pattern as the control mouse. (**D**) Representative image of an arginase + l-arginine-treated mouse, which shows no beneficial effect of l-arginine supplementation on the perfusion. (**E**) Representative live image of an l-citrulline-treated mouse, which also shows a comparable perfusion pattern as the control and l-arginine-treated mouse. (**F**) Representative image of an arginase + l-citrulline-treated mouse, which shows more perfused vessels per villus compared to arginase and arginase + l-arginine-treated animals.

To determine the effect of arginase injection on the microcirculation in one of the end organs, the microcirculation in the jejunal villi of arginase-treated mice was measured with the SDF-imager. Arginase infusion resulted in an impaired microcirculation, as deduced from a significantly lower total number of perfused vessels than in control mice (*p* < 0.05; Figure 9A and Figure 10A,B). l-arginine supplementation in arginase-treated mice did not result in improvement of the microcirculation, indicated by a similar total number of perfused vessels or number of perfused vessels per villus (Figure 9A,B and Figure 10D). On the contrary, l-citrulline supplementation in arginase-treated mice was associated with a ~85% higher total number of perfused vessels (Figure 9A,B and Figure 10F). In addition, the total number of perfused vessels in citrulline + arginase-treated mice did not differ from control mice supplemented with l-citrulline (Figure 9A).

MFI, the determination of the predominant type of flow in the villi in the four quadrants of the image, was significantly lower in arginase-treated than control mice (1.7 ± 0.1 *vs.* 2.9 ± 0.1, *p* < 0.001; Figure 5C). In addition, l-arginine supplementation did not result in a higher MFI in arginase-treated mice, while arginase-infused l-citrulline supplemented animals had a significantly higher MFI compared to arginase-infused animals alone (2.8 ± 0.1 *vs.* 1.7 ± 0.1, *p* < 0.001).

## 4. Discussion

To our knowledge, this is the first study comparing the acute effects of l-citrulline and l-arginine supplementation on tissue and systemic arginine availability, NO production and microcirculation in an acute arginine-deficient state. The present study provides new evidence that l-citrulline, but not l-arginine, supplementation improves the microcirculation in an end organ during conditions with arginase-induced acute arginine deficiency by increasing NO production. Our findings may have important consequences for further studies that aim to improve acute dysregulation of arginine metabolism, such as in sickle cell disease [34].

Previous studies have shown that increasing plasma arginine concentrations by supplementing l-arginine resulted in an increased arteriole diameter in the cremaster muscle of transgenic sickle mice [11,35]. In contrast, arginine supplementation did not result in an improved microcirculation or NO production in our previously developed septic model with a prolonged increase in arginase activity and accompanying arginine deficiency [17]. In agreement with our earlier data from the septic model, l-arginine supplementation in this study did not increase local arginine availability, NO production nor the intestinal microcirculation during acutely-enhanced plasma arginase activity.

In addition to sickle cell disease, conditions, such as endotoxemia, hemolysis, sepsis, asthma and liver diseases, are also characterized by an increased plasma arginase activity [2,8,22,36,37,38,39,40]. In these examples, arginase-induced arginine deficiency also resulted in decreased intracellular arginine availability for NO production due to a CAT-mediated exchange of intracellular arginine for extracellular high ornithine concentrations, resulting from the high plasma arginase activity [17,41,42]. l-citrulline was suggested as an alternative for enhancing intracellular arginine and NO concentrations, since citrulline is capable of bypassing the arginase activity in the gastro-intestinal tract [43,44]. This bypass effect was also observed in the present study, as the supplemented l-citrulline resulted in a >2-fold increase in plasma arginine concentrations during this acutely-enhanced arginase activity. The necessary intracellular or renal conversion of citrulline into arginine may be the underlying beneficial feature of citrulline supplementation *versus* arginine, as this allows an increase in arginine plasma and tissue concentrations during conditions with enhanced arginine catabolism. Indeed, l-citrulline supplementation also enhanced the ornithine concentrations during this study, which may be the result of the conversion of the circulating arginine derived from citrulline or the diminished conversion of ornithine into citrulline [45,46]. On the contrary, the supplemented l-arginine was converted into ornithine as observed in the increased plasma and tissue ornithine concentrations during the basal and experimental condition with enhanced arginase activity. Furthermore, the presence of argininosuccinate synthase (ASS) in endothelial cells allows the cells to use exogenous l-citrulline to increase intracellular arginine for NO production [47,48,49]. Thus, citrulline can act as a direct vasodilator in the microcirculation [50], which may contribute to the beneficial effects of l-citrulline supplementation on the microcirculation observed in this study. Another benefit of citrulline supplementation is the incomplete hepatic clearance of citrulline, resulting in enhanced citrulline availability from supplementation or endogenous synthesis in the gut, which can be used to enhance *de novo* arginine synthesis in the kidney [51,52] or in endothelial cells. Interestingly, citrulline supplementation resulted in an increased citrulline concentration in liver tissue, in agreement with a low affinity of the uptake of citrulline in liver. However, the intracellular citrulline concentrations in liver tissue were not as high as in renal tissue, which may be explained by an enhanced conversion of citrulline into ornithine and urea as part of the urea cycle.

In one non-controlled pilot study, citrulline enhanced arginine availability and relieved exertional fatigue and dyspnea in sickle cell disease [16]. Thus far, the influence of l-citrulline supplementation on the microcirculation in sickle cell disease remains to be investigated. We previously demonstrated that l-citrulline supplementation increased arginine availability and NO production during a prolonged arginine-deficient state, partly caused by an enhanced arginase activity [17]. In the present study, with an acute enhanced arginase activity, comparable results were observed with l-citrulline supplementation. As observed in this study, citrulline supplementation enhanced the arginine concentration in the kidney ~25-fold compared to control conditions, resulting in a significantly higher circulating arginine concentration than in arginine-supplemented animals. This positive enhancement of the plasma arginine concentration was also present in the arginase-treated group, which strengthens our hypothesis that citrulline may be the preferred substrate to increase the arginine availability in arginine-deficient conditions. In addition, gastrointestinal complaints after the intake of large l-arginine amounts are not observed in studies with large oral l-citrulline amounts [43,53,54,55]. Furthermore, in humans, l-citrulline supplementation is a good therapeutic strategy to combat splanchnic hypoperfusion-induced intestinal compromise in strenuous exercise, as this resulted in an increased number of perfused small sublingual vessels and prevention of splanchnic hypoperfusion and gastrointestinal damage [56].

Additional issues remain to be investigated. The microcirculatory measurements were conducted in the jejunal villi, but not in the liver or renal tissue of these animals, as a limitation of the SDF-imager in this model. The SDF-imager is applied on tissue surfaces, especially mucosa, and measures perfused vessels, as the 530-nm light is absorbed by the hemoglobin in red blood cells [57]. Measurements of the microcirculation in liver and renal tissue with the SDF-imager on the external surface [58,59] is less suitable in this study, as these tissues exhibit a large amount of red blood cells, which prevents the accurate measurement of the microcirculation in the tissues relevant for our study. To visualize the microcirculation in renal tissue [59], the retroperitoneal cavity has to be opened and part of the renal capsule removed, to visualize the renal tissue responsible for the arginine *de novo* synthesis. This not only results in tissue damage, leading to free-hemoglobin, but also a prolonged surgical procedure, which interferes with the fixed time period of the experiment. As previously observed in conditions with an acute increase in arginase, the gut microcirculation is the first organ system to derange, after which other microcirculatory beds are affected [33,60,61,62], which led us to the present experimental protocol, which only investigated the microcirculation in the jejunal villi. Therefore, due to technical limitations in this study, the influence of l-citrulline supplementation during an acute arginase-induced arginine deficiency in other vascular beds still needs to be examined. Another limitation of this study is that the effects of an acute arginase-induced arginine deficiency were not investigated in a mouse model of sickle cell disease. Transgenic mouse models to investigate sickle cell disease use mice exhibiting mild and severe pathology with different expression patterns of beta(S)-globin or hemoglobin-S, such as NY1DD mice and Berkeley (BERK) mice [10,11]. Future studies should determine the role of arginase in the acute hemolytic crises in these mice and the beneficial effects of l-citrulline supplementation.

## 5. Conclusions

In conclusion, our study showed that l-citrulline supplementation resulted in improved microcirculation during an arginase-induced acute arginine-deficiency state by increasing arginine availability and NO production, while these effects were not achieved with l-arginine supplementation.

## Supplementary Files

Supplementary File 1
