# Supplementary material for: Citrulline Supplementation Improves Organ Perfusion and Arginine Availability under Conditions with Enhanced Arginase Activity
Source: Nutrients. 2015 Jun 29;7(7):5217–38. doi: 10.3390/nu7075217 (PMC4516994; doi:10.3390/nu7075217)
Supplement: Supplementary File 1 [file nutrients-07-05217-s001.docx]

**Supplementary Information**

**Table S1.** Normalized amino acid values of l-arginine and l-citrulline supplementation during control and arginase-treated conditions.

| **Amino Acid** | **Arginine i.p.** | **Citrulline i.p.** | **Arginase i.p.** | **Arginase + Arginine i.p.** | **Arginase + Citrulline i.p.** |
| --- | --- | --- | --- | --- | --- |
| **Plasma** | | | | | |
| Arginine | 2.50 | 5.89 | 0.33 | 0.48 | 1.38 |
| Citrulline | 0.53 | 43.69 | 1.19 | 0.39 | 29.55 |
| Ornithine | 2.15 | 4.17 | 1.85 | 6.08 | 5.82 |
| Lysine | 1.01 | 1.45 | 2.09 | 1.04 | 1.04 |
| **Jejunal tissue** | | | | | |
| Arginine | 2.16 | 1.49 | 0.46 | 2.10 | 1.53 |
| Citrulline | 1.40 | 3.99 | 1.16 | 1.17 | 3.17 |
| Ornithine | 5.67 | 2.57 | 1.56 | 1.80 | 2.98 |
| Lysine | 10.03 | 3.39 | 1.57 | 3.83 | 4.21 |
| **Liver tissue** | | | | | |
| Arginine | 0.36 | 1.05 | 0.57 | 0.92 | 0.93 |
| Citrulline | 2.33 | 13.90 | 1.58 | 2.03 | 8.60 |
| Ornithine | 6.73 | 8.25 | 1.23 | 4.69 | 6.66 |
| Lysine | 0.54 | 1.97 | 0.85 | 3.70 | 1.78 |
| **Renal tissue** | | | | | |
| Arginine | 2.73 | 22.08 | 0.64 | 2.13 | 22.38 |
| Citrulline | 1.25 | 14.61 | 1.00 | 1.20 | 13.97 |
| Ornithine | 4.47 | 10.53 | 1.71 | 43.22 | 14.00 |
| Lysine | 1.77 | 2.65 | 0.72 | 0.73 | 2.76 |

Values are normalized for the control group, resulting in an increase or decrease of the presented amino acids compared to the control group per tissue and amino acid concentration; i.p., intraperitoneal.

© 2015 by the authors; licensee MDPI, Basel, Switzerland. This article is an open access article distributed under the terms and conditions of the Creative Commons Attribution license (http://creativecommons.org/licenses/by/4.0/).
